# Supermicrosurgery in Reconstructive Surgery: A Narrative Review

**DOI:** 10.1055/a-2822-5320

**Published:** 2026-05-29

**Authors:** Tae Hyung Kim, Jin Geun Kwon, Changsik John Pak, Hyunsuk Peter Suh, Joon Pio Hong

**Affiliations:** 1 Department of Plastic and Reconstructive Surgery, University of Ulsan, College of Medicine, Seoul Asan Medical Center, Seoul, South Korea

**Keywords:** supermicrosurgery, perforator flap, high-frequency ultrasound, superficial plane, robotic microsurgery

## Abstract

Supermicrosurgery, defined as the anastomosis of vessels smaller than 0.8 mm, represents a significant evolution in the field of reconstructive microsurgery. This review explores its technical foundations, clinical applications, and future directions, with a particular focus on lower extremity reconstruction. By utilizing perforator-to-perforator anastomosis and thin flap techniques, supermicrosurgery allows for reduced donor morbidity, minimized risk to major vessels, and improved aesthetic and functional outcomes. The integration of high-frequency ultrasonography and advanced microsurgical tools has enhanced preoperative planning and intraoperative precision. Despite a steep learning curve, supermicrosurgery is increasingly applied in oncologic, traumatic, ischemic, and diabetic foot reconstructions. Continued innovation in imaging, instrumentation, and robotic assistance suggests a promising future for this subspecialty.

## Introduction

Microsurgery has long been a cornerstone of complex reconstruction, built upon foundational principles established by pioneers such as Dr. Harry J. Buncke. As the field matured, limitations emerged, particularly in cases involving small-caliber vessels and anatomically constrained regions. 1 Supermicrosurgery—targeting vessels <0.8 mm—has emerged as a refined extension of conventional techniques. Initially developed in the context of lymphatic surgery, it now plays a central role in various reconstructive procedures, including extremity, head and neck, and breast reconstruction. 2 3 4 This approach is valued not only for the versatility and flexibility of flap application across different anatomical regions, but also for its ability to minimize donor site morbidity. 3 5 Its success relies on technical expertise, high-resolution imaging, and improved understanding of perforator anatomy. In this review, we examine the principles and instruments enabling supermicrosurgical precision, explore its clinical applications—particularly in lower extremity reconstruction—and highlight recent innovations in imaging and flap design. 6 7 Additionally, we address training challenges and speculate on future directions involving robotic assistance and bioengineered tools. Through this comprehensive review, we aim to provide a practical and forward-looking resource for clinicians and researchers engaged in microvascular reconstruction.

## Technical Principles and Microsurgical Tools

A fundamental principle in supermicrosurgery is the optimization of the surgical environment. A stable operating microscope with ergonomic support, adequate magnification, and coaxial lighting is crucial. The surgeon's posture, hand positioning, and instrument organization all contribute to microsurgical efficiency and fatigue reduction. 1 8 Vibration dampening, avoidance of drafts, and use of noise-free lighting are subtle but essential aspects of the working environment. Foot pedals for bipolar, microscope zoom, and microscope positioning must be ergonomically placed to avoid interruption during fine movements.

Training is a critical pillar of success in supermicrosurgery. The steep learning curve associated with manipulating submillimeter vessels necessitates dedicated practice using artificial vessels, live animal models, and simulation-based microsurgical courses. Key skills include tremor control, ambidexterity, consistent needle entry angles, and the ability to perform watertight anastomoses. 8 9 Assessment tools such as the Structured Assessment of Microsurgery Skills (SAMS) and video-assisted review are valuable for objective evaluation. 9

Preoperative planning begins with flap selection, perforator mapping, and recipient site evaluation. Supermicrosurgery leverages the use of small-caliber perforators as both donor and recipient vessels, and the use of high-resolution Doppler or ultrasound is indispensable in identifying their course, diameter, and flow pattern. 6 Planning also involves strategizing vessel orientation to prevent kinking, redundancy, or compression. Choice of flap—whether superficial circumflex iliac artery perforator (SCIP), anterolateral thigh (ALT), or another—must account for vessel size match and pedicle length to allow tension-free anastomosis. 7

Recipient vessel selection is a nuanced task, especially in ischemic or posttraumatic fields. Ideal vessels should be of appropriate caliber, show pulsatility or brisk venous return, and be in proximity to the defect while avoiding prior radiation or scarred fields. 6 10 Once selected, vessel preparation is done under high magnification with meticulous adventitial stripping using microforceps and fine scissors. Vasospasm should be managed by warm saline irrigation, topical lidocaine, papaverine, or gentle mechanical dilation.

Vessel dissection and preparation require careful attention to surrounding tissues to preserve collateral circulation. Dissection in the correct anatomical plane minimizes damage to the vasa vasorum and perivascular nerves. Hemostasis should be achieved without thermal spread; bipolar coagulation in low-voltage mode is preferred. During flap inset, careful pedicle orientation and avoidance of tension are essential. Technical errors at this stage can lead to thrombosis, congestion, or flap failure.

**Fig. 1:**
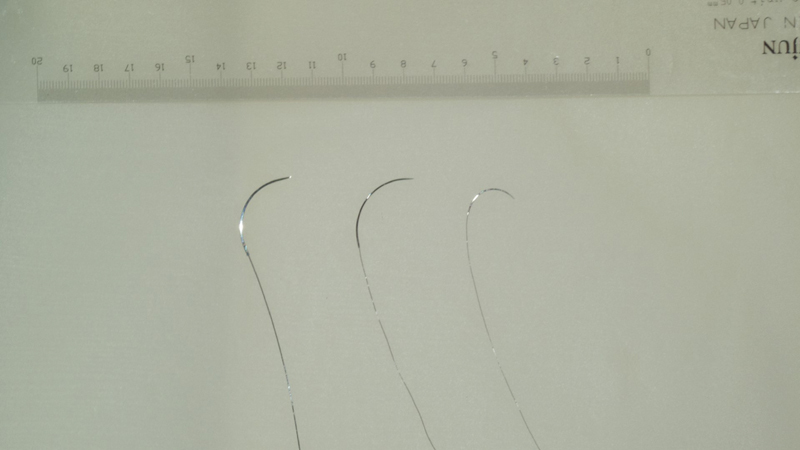
Comparison of micro nylon sutures: 10–0, 11–0, and 12–0 from the left to right.

Supermicrosurgery demands exceptional precision and specialized instruments. Suture materials such as 10–0, 11–0, and even 12–0 nylon with needles as fine as 30 μm in diameter are essential ( Fig. 1 ). These are handled with ultrafine forceps and needle holders with tip diameters ranging from 0.05 to 0.3 mm. Vascular clamps designed for vessels between 0.5 and 1 mm apply minimal pressure to avoid endothelial trauma. Coagulation is performed with bipolar systems using low-current settings to prevent thermal injury. High-magnification microscopes (up to 20 × ) and three-dimensional exoscopes facilitate ergonomics and co-surgeon access. Intraoperative fluorescence imaging, such as indocyanine green (ICG) angiography, aids in assessing perfusion. Intraoperative fluorescence imaging has become an indispensable adjunct in supermicrosurgery. In addition, high-frequency ultrasound could play an important role in supermicrosurgery in terms of planning and postoperative care.

## Clinical Applications in Lower Extremity

The lower extremity, with its complex vascular anatomy and high susceptibility to trauma and ischemia, has emerged as a prime region for the application of supermicrosurgical techniques. 3 10 The use of perforator-to-perforator anastomosis allows surgeons to bypass damaged or deeply situated major vessels, enabling more localized and less invasive reconstructions. 11 This strategy reduces operative time and broadens the spectrum of suitable recipient vessels, particularly in ischemic limbs or cases where conventional options are compromised.

**Fig. 2:**
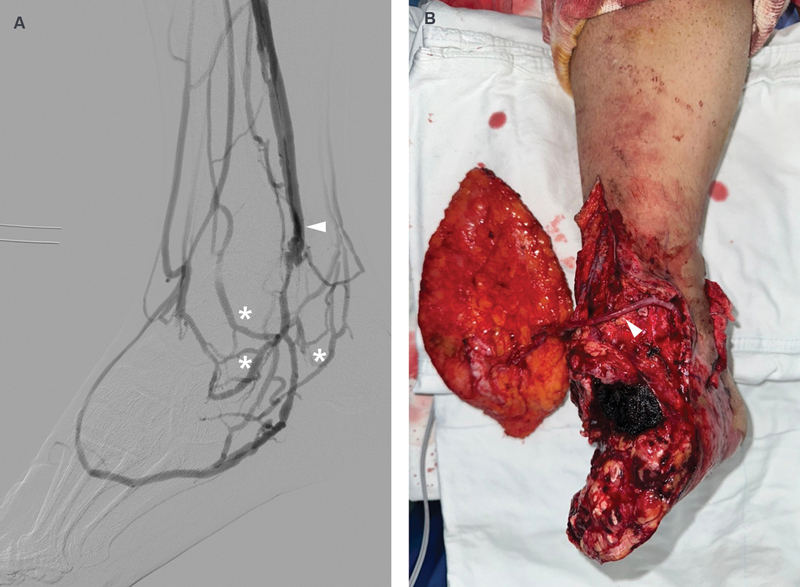
Clinical images of a 34-year-old male patient with Buerger's disease. ( **A** ) Angiographic image revealing collateral vessels (asterisk) and arterialized superficial veins (arrowhead). ( **B** ) Intraoperative image depicting the use of an arterialized vein (arrowhead) as a recipient vessel due to the absence of major arteries.

For example, in a 34-year-old male patient with Buerger's disease, traditional arterial routes were non-viable due to poor distal runoff ( Fig. 2 ). Instead, arterialized superficial veins were successfully used as recipient vessels, resulting in a healthy and viable flap 1 month postoperatively. This case illustrates the feasibility and effectiveness of supermicrosurgery in limb salvage, particularly when standard vascular access is limited.

**Fig. 3:**
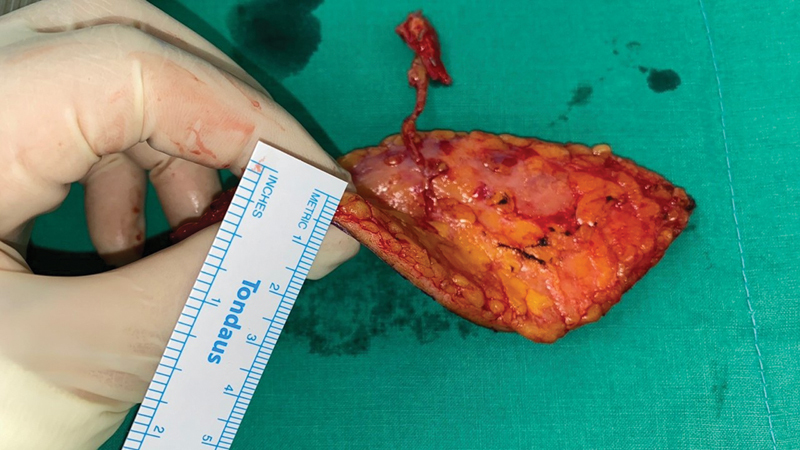
Intraoperative image of a 4 mm-thick ultrathin anterolateral thigh flap during elevation.

Similarly, in oncologic reconstruction, clean wound beds allow for reliable flap inset, and thin flaps become especially advantageous for both functional and aesthetic outcomes. The elevation of a 4-mm ultrathin ALT flap ( Fig. 3 ) demonstrates how meticulous flap design can be achieved using supermicrosurgical principles. Importantly, these thin flaps are not limited by the patient's body habitus; rather, successful elevation depends on accurate identification of the perforator's course through preoperative imaging and intraoperative planning. 12

Beyond simple vessel size, the goal of supermicrosurgery in lower extremity reconstruction is to facilitate aesthetic and functional limb salvage. The ability to use shorter pedicles, smaller perforators, and thinner flaps contributes to faster recovery and reduced donor site morbidity. 6 7 Moreover, these advantages are not confined to the lower extremity—facial and scalp units, with their demand for delicate contour and minimal tissue bulk, may benefit even more profoundly from the principles of supermicrosurgery. 13 In particular, the SCIP flap has shown excellent outcomes in facial reconstruction due to its redundancy, pliability, and naturally thin characteristics. 14 15

Thus, supermicrosurgery not only improves safety and versatility but also enhances aesthetic reconstruction and accelerates patient recovery. Achieving these outcomes requires a comprehensive understanding of flap elevation planes and the integration of high-resolution imaging tools, which together form the cornerstone of modern, physiology-driven reconstructive planning.

## Planes for Supermicrosurgery and Flap Thinning

Recent advances in flap elevation have emphasized anatomical planes that optimize both vascular reliability and flap thinness. 16 17 The concept of perforasomes, introduced in the early 2000s, supported the harvesting of thinner fasciocutaneous flaps based on reliable vascular territories. 18 The suprafascial and subdermal planes allow elevation of ultrathin and superthin flaps with reduced donor morbidity and improved contour. 7 16 Though partial necrosis remains a concern, especially in flaps exceeding 20 cm, improved anatomical understanding and precise dissection mitigate risks. 19 20 These thin flaps have demonstrated infection control and functional outcomes comparable to muscle flaps.

While supermicrosurgery is traditionally defined by the anastomosis of vessels smaller than 0.8 mm, its modern evolution is increasingly rooted in a deeper understanding of anatomical planes rather than vessel diameter alone. The shift toward perforator-based reconstructions naturally leads to greater interest in the course of perforators within the subcutaneous tissue. 2 16 21 As a result, the distinction between superficial and deep fascial planes becomes clinically significant. 17

This plane-based perspective allows for intentional flap thinning, either by elevating from the suprafascial or subdermal layers or by directly designing superthin flaps from the outset. Such approaches reduce unnecessary tissue bulk, optimize contour, and most importantly, minimize donor site morbidity. By aligning flap elevation with the vascular architecture confirmed via imaging or intraoperative dissection, surgeons can achieve both reliable perfusion and superior cosmetic results.

Thus, the core concept of supermicrosurgery has expanded: It is no longer merely about vessel size but also about the intelligent design of the flap plane. Mastery of these planes enables more versatile and physiologically tailored reconstructions, especially when dealing with functionally or cosmetically sensitive regions. The plane-centric paradigm underscores the need for precise ultrasonographic planning, microsurgical dexterity, and a nuanced understanding of tissue anatomy—all of which are essential for modern supermicrosurgical practice.

## Imaging Modalities

High-resolution imaging plays a pivotal role in supermicrosurgery by enabling precise identification of perforators, mapping vascular anatomy, and planning flap design. Each imaging modality offers distinct advantages depending on surgical needs and anatomical complexity.

CT angiography (CTA) provides a broad anatomical overview, making it especially effective for mapping deep and consistent perforators. 22 It has proven useful across a range of flap donor sites, including the ALT, SCIP, deep inferior epigastric artery (DIEA), superficial inferior epigastric artery (SIEA), latissimus dorsi, thoracodorsal artery perforator, superior gluteal artery perforator, and inferior gluteal artery perforator flaps. 22 23 24 25 26

Color duplex ultrasonography offers dynamic information about arterial flow and is particularly helpful in free flap planning. 24 27 In thin patients or in donor areas with variable anatomy, it provides greater clarity in selecting suitable perforators and assessing flow characteristics.

**Fig. 4:**
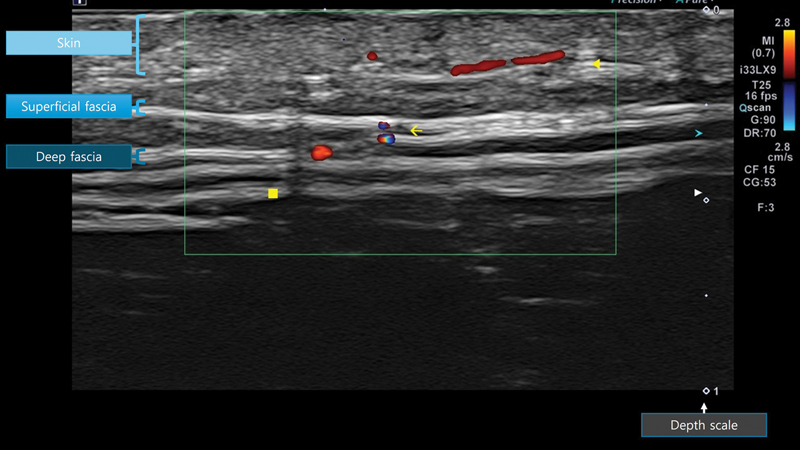
Perforator imaging with a 33 MHz probe. Imaging of the vascular course at the subdermal layer (arrowhead) superficial fascia perforating point (arrow), deep fat tissue (square).

High-frequency ultrasound (18–33 MHz) enhances preoperative planning by allowing real-time visualization of vessel caliber, trajectory, and depth. 28 Lower-frequency probes (∼5 MHz) are also useful, especially for identifying perforator location and the main pedicle in ALT flap design. Higher frequencies, on the other hand, provide superior resolution of superficial fascial and subdermal perforator pathways ( Fig. 4 ). This is especially beneficial for flap thinning and reducing donor site morbidity, as it allows surgeons to plan flaps according to precise anatomical planes.

Intraoperative ultrasonography extends imaging guidance into the surgical field. It effectively simulates the dissection process by visualizing fascial layers, muscle borders, and vascular structures, thus enhancing safety and accuracy during elevation.

Fluorescence imaging with ICG has emerged as an essential intraoperative tool for assessing tissue perfusion and vascular patency. 29 30 After intravenous injection, ICG binds to plasma proteins and fluoresces under near-infrared light. 31 This enables the surgeon to visualize microcirculation in real time via specialized camera systems. Unlike Doppler, which provides point-specific signals, ICG angiography generates a spatial perfusion map, enhancing intraoperative decision-making for flap trimming, perforator selection, and anastomosis validation.

ICG angiography typically takes around 60 to 90 seconds and integrates seamlessly into surgical workflows. 32 It can identify zones of marginal perfusion and detect early signs of congestion or ischemia, thereby reducing the risk of partial flap necrosis. In microsurgical training, ICG feedback is valuable for evaluating the quality of anastomosis by highlighting areas of leakage or delayed filling. These advantages make ICG a technical cornerstone of supermicrosurgery, complementing meticulous dissection, anatomical planning, and microvascular precision. 33

In summary, the combined use of CTA, duplex ultrasound, high-frequency probes, and ICG angiography enables a comprehensive and individualized approach to vascular imaging. This multimodal strategy not only enhances surgical planning and execution but also contributes to safer, more reliable reconstructive outcomes.

## Customized Approaches and Postoperative Considerations

Supermicrosurgery enables patient-specific strategies based on vessel size, location, and flow characteristics. In limbs with compromised major arteries, collateral vessels or arterialized superficial veins offer alternative recipient options. Proper recipient vessel selection is vital, especially in regions like the knee or ankle, where joint position can alter flow dynamics. 7 11 Understanding the relationship between limb posture and perfusion informs postoperative care and flap monitoring strategies. 34

This evolution in planning reflects a broader conceptual shift from the angiosome to the perforasome and, ultimately, to the physiologic concept of the “physiosome.” While angiosomes and perforasomes provided general vascular territory maps, they are inherently probabilistic. In contrast, the physiosome concept emphasizes individualized vascular anatomy and perfusion, based on real-time imaging and physiologic characteristics. This shift has allowed supermicrosurgeons to move beyond static anatomical maps toward truly customized reconstructive strategies. 3 7 16

High-frequency ultrasonography plays a critical role in this paradigm by enabling detailed visualization of perforators and their branching patterns in each individual patient. 24 28 For example, while the ALT flap is often considered a workhorse due to its high perforator prevalence, some patients may have poorly developed ALT perforators but instead exhibit well-formed anteromedial thigh branches. By detecting these variants preoperatively, the surgeon can select more reliable donor sites, avoid unnecessary exploration, and tailor flap design for optimal thickness and contour.

This patient-specific approach enables flap thinning when appropriate, leading to reduced donor morbidity and improved aesthetic outcomes. Furthermore, it allows for the preservation of major vessels, which can be especially beneficial in high-risk patients or those with future reoperation potential. Such precision in planning and execution exemplifies the future of supermicrosurgery—not merely as a technical feat, but as a philosophy of individualized, anatomy-driven, and functionally optimized reconstruction.

## Training, Challenges, and Future Directions

**Video 1** Robotic microanastomosis on a 0.6-mm-diameter vein performed by a student without any prior surgical experience. The robot assists with delicate control of the adventitial tissue and suturing.


A significant barrier to widespread adoption of supermicrosurgery is the steep and prolonged learning curve required to achieve consistent results at the submillimeter scale. Traditional microsurgical training has been supplemented by simulation-based curricula utilizing artificial vessels, high-magnification microscopes, and pulsatile perfusion systems. These platforms allow trainees to develop fine motor control, depth perception, and precise suture handling prior to performing live surgery.

Recent advancements in robotic-assisted microsurgery are reshaping the educational and technical landscape. Robotic systems such as MUSA (MicroSure) and Symani Surgical System (MMI) offer mechanical stability, motion scaling, tremor filtration, and enhanced dexterity. 35 36 An in vivo study using robotic-assisted anastomosis on rat femoral vessels demonstrated comparable patency, accuracy, and proficiency to handsewn techniques when performed by surgeons with 5 years of microsurgical experience. 37 Robotic assistance helps level the playing field, allowing less experienced surgeons to safely perform microsurgical anastomosis early in their training. 36

**Fig. 5:**
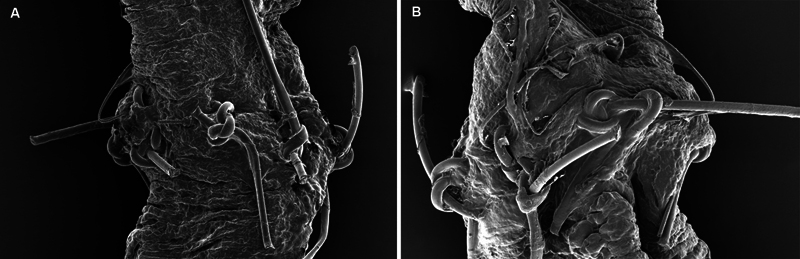
Scanning electron microscopy shows suture details on 0.4-mm vessels after robotic and manual microanastomosis. ( **A** ) Robotic microanastomosis demonstrates less suture loosening and breakage, with more consistent suture placement. ( **B** ) Manual suturing shows more frequent loosening and suture breakage.

Notably, emerging data from a soon-to-be-published multicenter study have demonstrated that the Symani system achieves significantly higher precision and patency rates than manual suturing in vessels averaging 0.6 mm ( Video 1 ). Furthermore, it showed comparable performance between novice and experienced microsurgeons after a brief adaptation period, effectively reducing the skill gap traditionally seen in microsurgery training. Interestingly, in anastomoses involving vessels smaller than 0.3 mm, the robot-assisted group not only achieved higher success rates but also demonstrated faster completion times ( Fig. 5 ).

These findings highlight the transformative potential of robotic systems not only for anastomosis but also for tissue dissection, vessel manipulation, and fine forceps application. As technology evolves, robotic supermicrosurgery is poised to expand its scope from lymphatic surgery and peripheral nerve coaptation to deeper, more anatomically constrained fields where traditional access is limited. With robotic instruments enabling high-resolution, angle-independent control, meticulous dissection in narrow or deep planes becomes achievable with unprecedented precision. 37 38

As robotic systems demonstrate feasibility in accurate anastomosis of vessels <0.3 mm, new frontiers for clinical application may emerge. For instance, in the context of neurodegenerative diseases—where vascular modulation and microcirculatory enhancement are under exploration—robotic supermicrosurgery could enable targeted, minimally invasive interventions. 39 Reduced operative trauma and improved reproducibility would translate to better outcomes and shorter recovery periods for patients.

In the long term, integration of artificial intelligence with robotic systems holds the potential for semiautomated or fully autonomous microanastomosis. This would not only improve consistency and reduce operator fatigue but also open the door to scalable microsurgical solutions in high-volume or remote settings. As such, robotic training is becoming an indispensable component of the supermicrosurgical curriculum and will likely define the next generation of precision reconstruction.
